# Prevalence of Brain Incidental Lesions Detected by ^68^Ga-DOTA Peptides PET/CT

**DOI:** 10.3390/medicina58070916

**Published:** 2022-07-10

**Authors:** Domenico Albano, Giorgio Treglia, Francesco Dondi, Francesco Bertagna

**Affiliations:** 1 Nuclear Medicine Department, ASST Spedali Civili of Brescia, 25123 Brescia, Italy; f.dondi@outlook.it (F.D.); francesco.bertagna@unibs.it (F.B.); 2 Department of Medical and Surgical Specialties, Radiological Sciences, and Public Health, Nuclear Medicine, University of Brescia, 25123 Brescia, Italy; 3 Clinic of Nuclear Medicine, Imaging Institute of Southern Switzerland, Ente Ospedaliero Cantonale, 6501 Bellinzona, Switzerland; giorgio.treglia@eoc.ch; 4 Department of Nuclear Medicine and Molecular Imaging, Lausanne University Hospital, University of Lausanne, 1011 Lausanne, Switzerland; 5 Faculty of Biomedical Sciences, Università della Svizzera Italiana, 6900 Lugano, Switzerland

**Keywords:** incidentaloma, PET/CT, nuclear medicine, meningioma, ^68^Ga-DOTA, neuroendocrine, somatostatin

## Abstract

*Background and Objectives*: ^68^Ga-DOTA peptides positron emission tomography/computed tomography (PET/CT) is usually applied for the study of neuroendocrine tumours, but other tumours such as meningioma may also have an increased radiopharmaceutical uptake. The aim of this retrospective study was to establish the prevalence and the meaning of brain incidental uptake among patients who performed ^68^Ga-DOTA peptides PET/CT for other reasons. *Materials and Methods*: Overall, 510 ^68^Ga-DOTA peptides PET/CT scans performed between January 2018 and February 2022 from 430 patients were reviewed for the analysis of incidental brain radiopharmaceutical uptake. All brain incidentalomas were compared with brain magnetic resonance imaging (MRI) and/or contrast-enhanced CT performed within an average time interval of ±60 days from PET/CT scan. *Results*: A total of 48 patients (14%) presented incidental focal intracranial radiotracer uptake. Thirty-eight (11%) of them had a suspected meningioma confirmed by MRI or contrast-enhanced CT imaging features. The remaining 10 had a final diagnosis different from meningioma (5 as brain metastases and 2 as venous anomalies) or were lost during the follow-up without performing MRI (*n* = 3). The average maximal standardized uptake value (SUVmax) of the suspected meningioma was 16.5 (range 5–33), and the average lesion to brain SUVmax ratio was 351 (range 80–550). *Conclusions*: Brain incidental uptake from the ^68^Ga-DOTA peptides PET/CT is not so rare, and meningioma is the most frequent cause.

## 1. Introduction

Incidentalomas are defined as unexpected and asymptomatic findings discovered during the course of examinations performed for other reasons and related to other organs. These findings usually are incidentally detected by several imaging examinations, such as ultrasonography, computed tomography (CT), magnetic resonance imaging (MRI), or positron emission tomography (PET), with different radiopharmaceuticals [1,2,3]. The detection of these incidentalomas may be crucial because they can trigger additional medical care, including unnecessary, sometimes invasive and expensive diagnostic tests. In nuclear medicine, the most common radiotracer used for PET imaging is fluorine-18-fluorodeoxyglucose (^18^F-FDG), and incidental findings with this radiotracer are quite common [4,5,6]. However, with PET, using other radiopharmaceuticals, such as radiolabelled PSMA or choline, the detection of incidental lesions is possible [7,8]. In contrast, with DOTA peptides tracers, these detections are very rare [9]. Meningiomas are quite frequent in findings, being the most common primary tumours of the central nervous system with an incidence of approximately 30% [10]. They are considered benign tumours arising from the meningeal layers, often asymptomatic and of small size. Meningiomas are often discovered incidentally during routine imaging of the brain or spine, but in some cases, large lesions or those localized in critical positions (such as skull base or cerebellopontine angles) may be symptomatic, causing mass-effect and neurological disorders [11]. ^68^Ga-DOTA peptides are radiotracers that are routinely used in PET imaging for the diagnosis and staging of neuroendocrine tumours (NETs), especially gastroenteropancreatic (GEP) NETs, due to their ability to introduce somatostatin (SST) receptors, which are overexpressed in these tumours [12]. However, meningiomas also have a high expression of SST receptors such as NETs [13]. Thus, PET with ^68^Ga-labelled somatostatin analogues may be used for early diagnosis, restaging, and the selection of peptide receptor radionuclide therapy (PRRT) in these tumours [13]. Besides meningioma, other potential causes of brain uptake are described in the literature, such as brain metastases or venous abnormalities.

There are limited data available in the literature on the prevalence of incidental brain uptakes, especially meningiomas, in patients who performed ^68^Ga-DOTA peptides PET [14,15]. Due to the properties of ^68^Ga-DOTA peptides PET/CT [13], our aim was to investigate the rate of brain incidental uptake detected by ^68^Ga-DOTA peptides PET/CT in a large population and their nature.

## 2. Materials and Methods

We have retrospectively evaluated 430 patients who underwent ^68^Ga-DOTA peptides PET/CT for the evaluation of NETs, both for staging or restaging purposes, from January 2018 to February 2022 for a total of 510 scans. The mean age ± standard deviation was 62 ± 15 (range 33–81 years). There was a small prevalence of females (*n* = 220) compared to males (*n* = 210). The most common tumours studied were GEP NET (274 cases), followed by lung carcinoid (45 cases) and searching of primary unknown neuroendocrine lesion (39 cases). The primary indication was the restaging of disease, followed by staging, diagnosis and selection for PRRT (Table 1). An average activity of 161 MBq (range 112–197 MBq) of ^68^Ga-DOTATOC (*n* = 300) or ^68^Ga-edotreotide (*n* = 210) was administered intravenously, and PET/CT was acquired about 60 min after injection on a Discovery ST or 690 PET/CT scanners (General Electric) with standard CT parameters (80 mA, 120 kV without contrast; 3 min per bed-PET-step of 15 cm). The reconstruction was performed in a 256 × 256 matrix and 60 cm field of view. On a Discovery 690 tomograph, time of flight and point spread function algorithms were used for the reconstruction of images, with a filter cut-off of 5 mm, 18 subsets and 3 iterations. For Discovery ST tomographs, an ordered subset expectation maximization (OSEM) algorithm with a filter cut-off of 5 mm, 21 subsets, and 2 iterations was used. The images were acquired from the mid-thigh to the vertex and were analysed visually by searching any focal brain uptake with activity higher than the background. Then, any brain focal uptake was analysed semi-quantitatively by measuring the maximum standardized uptake value (SUVmax); SUVmax was calculated within a volume of interest drawn by visual assessment around the suspected brain lesion on PET/CT. Moreover, the ratio of SUVmax of brain lesion/SUVmax of normal brain parenchyma was calculated in all cases. The SUV of normal brain parenchyma was measured at the occipital region. All these analyses were conducted by an expert nuclear medicine physician (DA). For a confirmation of the nature of brain incidental uptake, the suspicious areas of high tracer uptake in the brain were then compared with brain MRI or contrast-enhanced CT imaging. They were performed within a mean interval of ±60 days from a PET/CT scan (range 3–100 days).

### Statistical Analysis

The numeric variables were described as mean, standard deviation, minimum and maximum. The descriptive analysis of categorical variables comprised the calculation of simple and relative frequencies. The prevalence of brain incidental lesions and meningioma was calculated as follows: prevalence = number of patients with brain incidental uptake or meningioma/number of patients evaluated with PET/CT × 100. The statistical significance of the continuous variables was tested with Student’s *t*-test. MedCalc (Belgium) was used as software for statistical analysis.

## 3. Results

**Figure 1:**
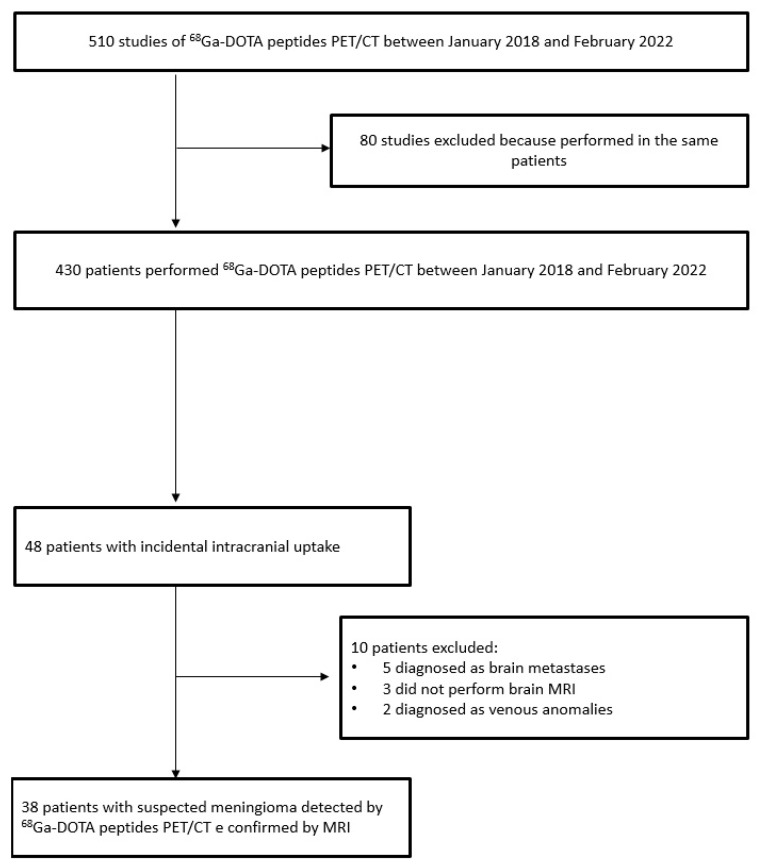
Flow chart of patients included in the study.

A total of 510 ^68^Ga-DOTA peptides PET/CT scans in 430 patients were performed between January 2018 and February 2022. A total of 48 patients (11%) had focal intracranial radiotracer uptake. Thirty-eight (9%) out of the forty-eight had a suspected meningioma confirmed by MRI imaging features. The remaining 10 were finally diagnosed as non-meningiomas considering MRI features: 5 as brain metastases from GEP NETs, 2 as venous anomalies, and 3 without MRI diagnoses; thus, the lesion confirmation was not available (Figure 1). The main clinical and epidemiological features (age, gender, size) were not significantly different between meningioma and brain metastases. Moreover, three of five brain metastases were intra-axial, and the remaining two were extra-axial. In all penitents with brain metastases, the brain lesion was isolated.

A sub-analysis for radiotracer (^68^Ga-DOTATOC and ^68^Ga-edotreotide) had a similar detection rate: among 300 ^68^Ga-DOTATOC PET/CT scans, incidental brain uptake was registered in 27 cases (9%), while among 210 ^68^Ga-edotreotide PET/CT scans, there were 21 cases (10%).

The main features of these 38 patients with suspected meningioma confirmed by MRI are reported in Table 2.

**Figure 2:**
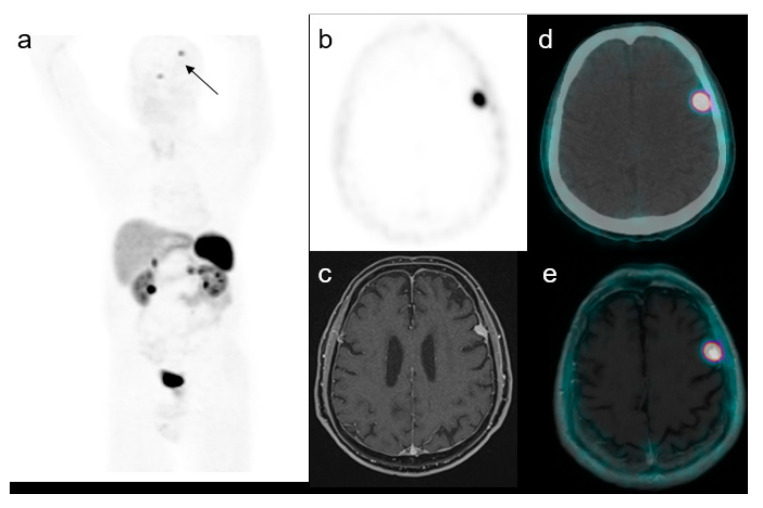
A representative case of a 59-year-old male with an increased uptake in the brain on the left side at maximum intensity projection (MIP, (**a**)). Axial PET (**b**), MRI (**c**), PET/CT fused (**d**) and PET/MRI fused images (**e**) confirmed the presence of a focal uptake in the left frontal region with a SUVmax of 17.5 confirmed to be a meningioma.

**Figure 3:**
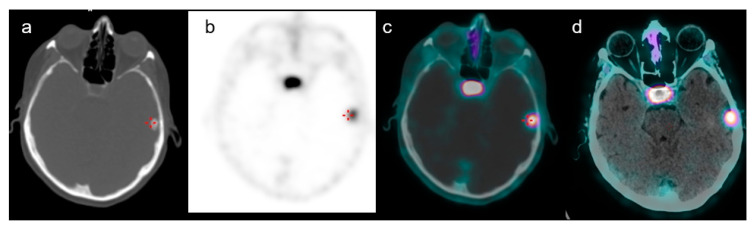
A case of a 66-year-old female with a history of GEP-NET who performed ^68^Ga-DOTATOC PET/CT for restaging purposes. Axial CT of PET (**a**), PET (**b**), PET/CT fused images (**c**) and PET/contrast-enhanced CT-fused images (**d**) confirmed the presence of a focal uptake in the left frontal region with a SUVmax of 9. Subsequent histological examination confirmed meningioma.

The average age was 66 years (range 45–75); females were 20 and males were 18. The mean lesion size was 15 mm (range 7–30 mm). In 4 out of 34 patients, two brain incidental lesion uptakes were registered. Twenty-one (50%) brain incidental uptakes were localized on the right side, which is the same number as on the left side. The frontal and temporal regions were the most frequent sites, with 16 (38%) and 13 (31%) lesions, respectively. The mean SUVmax of the suspected meningioma was 16.5 (range 5–33), and the lesion to brain SUVmax ratio was 351 (range 80–550) (Figure 2 and Figure 3). The DOTA uptake of meningioma expressed as SUVmax, and lesion to brain SUVmax ratio was not significantly different compared to the brain metastases and venous anomalies (*p* = 0.657 and *p* = 0.508, respectively). The mean SUVmax and lesion to brain SUVmax ratio of the five brain metastases from GEP NET were 17.7 (range 7–43) and 355 (range 79–580), and two of the venous anomalies were 11 and 286 in one case, and 17.7 and 366 in the other.

## 4. Discussion

Historically, brain CT and MRI were considered the best imaging methods for the study of meningioma, diagnosis, treatment planning and post-therapy follow-up. However, CT and MRI may have some limitations that limit their accuracy [16], such as the difficulty to determine the exact boundaries of the lesions and the microscopic extension beyond the visible anatomical margins, or the difficulty to discriminate between residual disease and post-therapeutic changes after surgery or radiotherapy, or the impossibility to evaluate tumour grade.

Moreover, PET/CT with ^18^F-FDG, which is the most widely nuclear medicine tool used, presents several limitations for the detection of meningioma. This technique evaluates the uptake of a radiolabelled study-increased glucose analogue as an indirect sign of proliferation activity. The limitations include a low lesion to background uptake ratio, due to the fact that the brain has inherently high physiologic ^18^F FDG activity that reduces the sensitivity for meningioma detection and delineation [17].

For these reasons, new radiopharmaceuticals were introduced, and among them, ^68^Ga-DOTA peptides have emerged as a class of radiotracers that may be useful in the evaluation of meningiomas, because these tumours have an increased expression of SST receptors [18]. The lesion-to-background ratio with ^68^Ga-DOTA peptides is very high due to the low/absence uptake in the brain parenchyma and skull bones. A potential limitation could be the detection and delineation of lesions close to the sella and skull base due to the high physiological radiotracer avidity of the pituitary gland.

In our analysis, we found 48 brain incidental uptakes in the ^68^Ga-DOTA peptides PET performed for the evaluation of NETs, and 38 out of 48 were considered probable meningioma, with an overall prevalence of 11%. In all cases, the diagnosis was confirmed by an MRI scan. Our prevalence of brain incidental lesions in the ^68^Ga-DOTA peptides PET is slightly higher than those suggested by previous authors’ studies [14,15]. Cleary et al. [14] found a prevalence of 6.7% of brain incidental lesions investigating 313 patients who underwent ^68^Ga-DOTATATE PET/CT. Instead, Parghane et al. [15] found an even lower prevalence (smaller than 1.2%), with only 6 cases in 500 metastatic NETs performing ^68^Ga-DOTATATE PET/CT. These values of prevalence are very wide in range, probably due to the heterogeneity of the patients included [14,15].

Notably, we used different radiolabelled somatostatin analogues compared to the previous published studies (with potentially different affinities for SST receptors): this should not be the reason for this difference in the prevalence of brain incidental uptakes, but specific studies based upon different DOTA-peptide comparisons in this field are lacking.

According to previous data, we demonstrated that the majority of brain incidental uptakes in ^68^Ga-DOTA peptides PET were meningiomas. This finding is not surprising considering the high frequency of this brain tumour and its ability to take up the radiolabelled somatostatin analogues due to the high SST receptor expression. The prevalence of meningioma detected by PET seems to be higher than that revealed by MRI in a recent meta-analysis [19], where a value of 0.52% is reported. Conversely, regarding radiotracer uptake intensity, our data are quite similar to those in a previous study [15], where average SUVmax values of brain lesions were 17, and lesion-to-brain SUVmax ratio values were 340, while in our analysis, their values were 16.5 and 351, respectively. Instead, Cleary et al. [14] demonstrated lower values of SUVmax with a value of 4.9, while the lesion-to-brain SUVmax ratio was not calculated. This difference may be explained by the lack of histological diagnosis of all cases (with the impossibility to know the histological features, such as typical or atypical meningiomas usually characterized by different SST expression nature).

With regards to brain incidental uptake with PET using other radiotracers, Baiomy et al. [20] studied patients with recurrent prostate cancer who performed 850 ^18^F-fluciclovine PET/CT scans reported and found only 14 cases (1.6%) of incidental brain incidental uptake, and 10 cases (1.1%) were finally confirmed as meningiomas. This rate is lower than that derived in our paper but with a different radiotracer and with different uptake mechanisms. Meningiomas can also be incidentally detected by radiolabelled choline PET, as demonstrated by some case reports [21,22], without clear prevalence according to the literature data.

Aside from meningiomas, ^68^Ga-DOTA peptides brain uptake may have other causes or different meanings. First, they may be related to metastases of NETs tumours (even if they are less frequent than meningioma) and rarely to venous abnormality alterations. Regarding venous abnormalities, a possible explanation could be that the vascular endothelial cells have high expression of SST-2 receptors. Thus, several venous physiological patterns could have an increased uptake [23]. Although MRI is traditionally considered the gold standard for the detection of meningioma and its treatment planning, ^68^Ga-DOTA peptides PET may be more effective [24,25]. Rachinger et al. [24] derived a sensitivity of 90% of PET in discriminating between tumour tissue and tumour-free tissue, whereas MRI achieved 79% of sensitivity. Afshar-Oromieh et al. [25], in a series of 134 patients, demonstrated a better sensitivity of PET than MRI, detecting 19 additional lesions.

An open question is whether the PET acquisition for ^68^Ga-DOTA PET could be extended to all brains. Despite the low prevalence of brain uptake reported in this study, considering the absence of physiological brain ^68^Ga-DOTA uptake (as opposed to ^18^F-FDG), it could be useful to include the whole brain in the scan with the aim to recognize any focal uptake.

One limitation of our data is the absence of histological confirmation of meningioma in all patients; of course, this is due to the impossibility (and ethics) of undergoing histological analysis in patients with suspected non-aggressive brain lesion. Certainly, other central nervous system tumours may have an increased expression of SST receptors, such as NET brain metastases, haemangioblastomas, pituitary adenomas, medulloblastoma or gliomas [26]. However, these brain lesions usually have peculiar morphological features and uptake patterns on PET, such as the localization of lesions, which, in our study, were in the peripheral brain in the area of the meninges between cortex and bone, which is typical of meningioma. Another limitation is the relatively low sample of patients analysed, which is also due to the low prevalence of brain incidental uptakes. In our study, we focused upon the PET features, and we calculated the prevalence of PET incidental uptakes, not considering specifically CT features; this is due to the fact that CT and PET were performed without contrast and low resolution (CT with attenuation purpose), so with a low accuracy and resolution power. In future studies, PET/MRI scanners might be useful for the study of meningioma, using the advantages of both techniques in a unique scan.

## 5. Conclusions

In conclusion, with this study, we have demonstrated that brain focal incidental brain uptake in ^68^Ga-DOTA peptides PET/CT is not rare, being a potentially significant diagnostic reality that requires further investigation. Among these incidentalomas, meningioma is the most frequent finding. Due to the prevalence of these incidental findings, we also suggest including the brain regions during the acquisition of ^68^Ga-DOTA peptides PET/CT.

## Figures and Tables

**Table 1:** The main characteristics of 430 patients studied.

	Mean ± SD (Range)	*N* (%)
Age (years)	62 ± 15 (33–81)	
Sex male		210 (49%)
Sex female		220 (51%)
Site of primary NET		
GEP		274 (64%)
Lung		45 (10%)
Unknown		39 (9%)
Paraganglioma		26 (6%)
Pheochromocytoma		22 (5%)
Neuroblastoma		8 (2%)
Medullary thyroid carcinoma		6 (1%)
Insulinoma		5 (1%)
Meningioma		5 (1%)
Grading WHO GEP-NET		
G1		200 (73%)
G2		71 (26%)
G3		3 (1%)
Indications		
Search of primary lesion		55 (13%)
Staging		135 (31%)
Restaging		189 (44%)
PRRT selection		51 (12%)

SD: standard deviation; GEP: gastroenteropancreatic; NET: neuroendocrine tumour; PRRT: peptide receptor radionuclide therapy.

**Table 2:** The main features of 38 patients with incidental brain uptakes confirmed as meningioma.

	Mean ± SD (Range)	*N* (%)
Age (years)	66 ± 18 (45–75)	
Sex male		18 (47%)
Sex female		20 (53%)
Site of primary NET		
GEP		20 (53%)
Lung		7 (18%)
Unknown		3 (8%)
Paraganglioma		5 (13%)
Pheochromocytoma		2 (5%)
Neuroblastoma		1 (3%)
Grading WHO GEP-NET		
G1		16 (80%)
G2		3 (15%)
G3		1 (5%)
Indications		
Search of primary lesion		6 (16%)
Staging		6 (16%)
Restaging		20 (52%)
PRRT selection		6 (16%)
Lesion size (mm)	15 ± 6 (7–30)	
Multifocal brain uptakes		4 (11%)
Location		
Right frontal region		9 (21%)
Left frontal region		7 (17%)
Right temporal region		6 (15%)
Left temporal region		7 (17%)
Right parietal region		5 (12%)
Left parietal region		5 (12%)
Left cerebellum		1 (2%)
Right cerebellum		1 (2%)
Left parasellar region		1 (2%)
SUVmax	16.5 ± 3.7 (5–33)	
Lesion to brain SUVmax ratio	351 ± 198 (80–550)	

SD: standard deviation; GEP: gastroenteropancreatic; NET: neuroendocrine tumour; PRRT: peptide receptor radionuclide therapy; SUV: standardized uptake value.

## Data Availability

Data are not public, but are present in our institution.
